# Reconstruction of Average Subtracted Tubular Regions (RASTR) enables structure determination of tubular filaments by cryo-EM

**DOI:** 10.1016/j.yjsbx.2020.100023

**Published:** 2020-03-09

**Authors:** Peter S. Randolph, Scott M. Stagg

**Affiliations:** a Institute of Molecular Biophysics, Florida State University, Tallahassee, FL 32306, USA; b Department of Chemistry and Biochemistry, Florida State University, Tallahassee, FL 32306, USA

**Keywords:** Electron microscopy, Lipid tubules, Protein filaments, Helical

## Abstract

As the field of electron microscopy advances, the increasing complexity of samples being produced demand more involved processing methods. In this study, we have developed a new processing method for generating 3D reconstructions of tubular structures. Tubular biomolecules are common throughout many cellular processes and are appealing targets for biophysical research. Processing of tubules with helical symmetry is relatively straightforward for electron microscopy if the helical parameters are known, but tubular structures that deviate from helical symmetry (asymmetrical components, local but no global order, etc) present myriad issues. Here we present a new processing technique called Reconstruction of Average Subtracted Tubular Regions (RASTR), which was developed to reconstruct tubular structures without applying symmetry. We explain the RASTR approach and quantify its performance using three examples: a simulated symmetrical tubular filament, a symmetrical tubular filament from cryo-EM data, and a membrane tubule coated with locally ordered but not globally ordered proteins.

## Highlights

• Tubules (filaments, membrane tubules, etc) that stray from perfect symmetry or have decorations can be difficult to process.
• New method Reconstruction of Average Subtracted Tubular Regions (RASTR), provides a way to isolate small areas of tubular architecture.
• Upweighted and masked areas can be treated as single particles and the structure can be resolved using conventional refinement.
• Successfully reconstructed protein filaments and membrane tubule decorations.

## Graphical abstract

**Figure:**
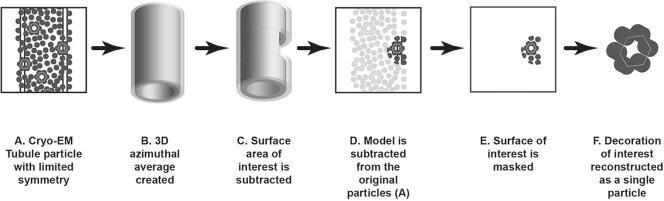

## 1 Introduction

Recent technological innovations have significantly increased the efficiency and resolution of structures determined by cryogenic electron microscopy (cryo-EM), causing a proliferation in the number of structures resolved with ever-increasing resolution (Callaway, 2015, Egelman, 2016, Glaeser, 2019, Nogales, 2015a). As many of the “low hanging fruit” structures have been determined, studies have shifted toward increasingly complex challenges, pushing the capabilities of current processing software, and many requiring novel methodologies (Ilca et al., 2015). Here we present a new approach called Reconstruction of Average Subtracted Tubular Regions (RASTR) which is designed to examine asymmetric architectural features of tubular structures.

Tubular architecture is common throughout all domains of life, as it is advantageous for a variety of functions. The large surface area to volume ratio can provide a platform for scission/fusion, gated compartments, reaction surfaces, etc (Mollenhauer and Morré, 1998, Shibata et al., 2009, Soulavie and Sundaram, 2016). In addition, tubules can form transport networks, allowing directed transport to distant structures/areas (Kudryashev et al., 2015, Sowinski et al., 2008), and be used for motility (Bardy, 2003, Craig et al., 2019). Tubules are usually formed by one of three processes: 1) helical arrangements of proteins (polymers) (Dominguez and Holmes, 2011). 2) self-assembly of lipid tubules when the appropriate lipids or small molecule(s) are present (Roux, 2013), 3) deformation of membranes through protein insertion (Roux, 2013, Simunovic et al., 2013).

Membrane tubules can form by perturbation of lipids bilayers through either small molecules/unique lipid headgroups (Jones et al., 2018, Spector et al., 1996) or insertion of proteins (Hariri et al., 2014, Liu et al., 2008, Simunovic et al., 2013). Lipids commonly form bilayers, micelles, or vesicles, but the insertion of proteins chains (commonly α-helices) causes an increase in surface area for one leaflet of the bilayer, forcing the architecture to deform into a variety of shapes including tubules (Stachowiak et al., 2010). Lipid tubules make up several intracellular organelles, such as the endoplasmic reticulum and trans-Golgi network. Their high curvature and large surface area to volume ratio make them ideal for protein accumulation and inter-organelle transport (Mollenhauer and Morré, 1998). Many proteins that perturb membrane structure form an ordered array (similar to a 2D lattice), which allows for uniform dimensions of the tubule and permit easier reconstruction of the structure. Some proteins (Sar1 tubules (Hariri et al., 2014, p. 1)) appear to only form small areas of local order, which limits the methods of resolving the architecture.

Helical polymers have been targets for electron microscopy since the technique’s inception (De Rosier and Klug, 1968). Resolving the structures of simple helical complexes can be relatively straightforward (He and Scheres, 2017), but structures lacking in helical symmetry, with bends/imperfections, or asymmetric components still present numerous issues. The first resolved structure containing helical architecture is the Bacteriophage T4 tail (De Rosier and Klug, 1968). Since then improvements in technology have allowed for a drastic increase in resolution, with a recent helical structure being determined to 1.9 Å (Schmidli et al., 2019). When the repeating units of a helical filament are displaced from the helical axis, they form a tubule. Helical tubules are abundant in biopolymers, and some well-known ones include microtubules, dynamin, and many bacterial pili. Originally, the structures of helical tubules have been solved by treating the tubules as helical crystals using the Fourier-Bessel approach (Diaz et al., 2010). However, the challenges of separating Bessel functions and Bessel overlap can make unambiguous determination of the helical symmetry for these specimens difficult, especially for tubules of large diameter (Crowther et al., 1985, Egelman, 2010). As the field has advanced, multiple techniques have been developed to deal with structures whose architecture does not conform to perfect symmetry. One popular approach is iterative real-space helical reconstruction (IRSHR) (Egelman, 2000). IRSHR separates the helical filament into small segments which are then treated as single particles. The segments are aligned, and a 3D model is generated before helical symmetry is determined and then applied. This allowed IHRSR to deal with a variety of issues that plagued the Fourier-Bessel approach for less than ideal samples. An advantage of helical symmetry is that each micrograph will contain all possible views of the particle (unlike 2D crystals). Additionally, once the helical parameters are known and applied, helical filaments are much easier to align than single particles and can get to a higher resolution than aligning without symmetry. Unfortunately, this process can be a double-edged sword, as helical symmetry increases the resolution for symmetrical elements of the filament but blurs out any asymmetric features. This becomes an issue if trying to capture asymmetric tubule decorations, such as microtubule-associated proteins (MAPs) (Nogales, 2015b, Wakefield et al., 2018).

**Fig. 1:**
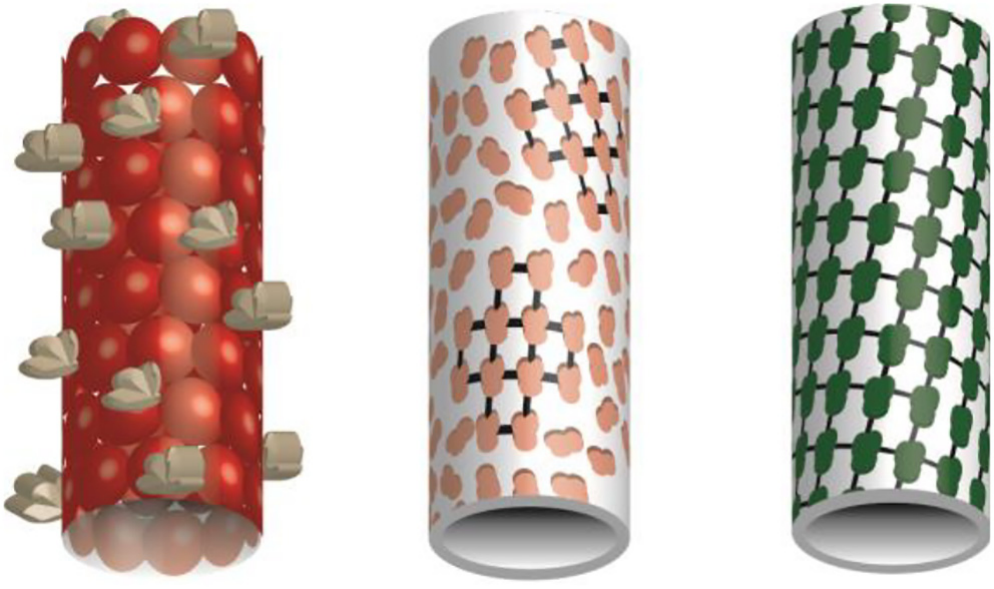
Tubule examples. Helical filaments with decorations (left). Membrane tubule with decorating particles with areas of local order (no global order) (middle). Tubule decorated with particles with global order, either a membrane tubule with globally ordered protein or a protein filament (right).

We have developed a technique for upweighting a section of the surface of tubular specimens in order to resolve their structures without applying symmetry. This has the potential to enable the structure determination of nonhelical decorations on helical tubules, tubular assemblies that are only locally ordered, and tubules with ambiguous symmetry (Fig. 1). Our technique, called Reconstruction of Average Subtracted Tubular Regions (RASTR), combines multiple electron microscopy processing tools to isolate a section of the tubule and upweight one side. Once sections are isolated and upweighted using RASTR, they are then treated as single particles and aligned and reconstructed. Here we present the RASTR process, and reconstructions of a helical filament from ideal and experimental data, without imposing symmetry.

## 2 Materials and methods

### 2.1 Specimen preparation

GalCer Tubes: Lipid nanotubes with a uniform diameter (25 nM) were constructed with molar equivalents of D-galactosyl-β-1,1′N-nervonoyl-D-erythro-sphingosine (GalCer) and a lipid mix (55% DOPC, 35% DOPS, and 10% cholesterol by molarity). GalCer and lipids were dissolved in chloroform which was dried under argon and rehydrated in a minimal salt buffer (25 mM HEPES pH 7.2, 1 mM Mg(OAc)_2_) to a final concentration of 0.8 mg/ml by vortexing. Tubes were assessed through negative stain TEM (2% Uranyl acetate) on a CM120 Biotwin and then cryogenically frozen with a FEI Vitrobot on c-flat holey carbon grids (2/2 300).

Generating ideal sample data: Sample data was generated from the VipA/VipB model from Kudryashev, et al (EMPIAR-10019) (Kudryashev et al., 2015). The VipA/VipB filament was chosen because of the high-resolution model, the readily available experimental data, and the high degree of symmetry. The pdb model (3J9G) was elongated to span the length of the box in pymol (Schrödinger, 2015) utilizing symmetry and alignment, then a 3.5 Å map was created with pdb2mrc from the EMAN suite. The map was projected using a sample star file containing ctf’s from the VipA/VipB experimental data, randomized phi, theta = ±90° (generate tubes with opposite polarities), psi = 90°, and x, y shifts = 0, creating a stack with box size 240x240 and 2 Å per pixel. No noise was given for the generated ideal data. Both the experimental data (available from EMPIAR) and the generated ideal sample data were used.

Generating low resolution initial models: Low resolution maps for the VipA/VipB were created from the original pdb using MolMap in the Chimera suite (Pettersen et al., 2004) from the extended pdb mentioned above. The maps were masked to the upweighted region of interest using an in-house script.

Sar1⋯GalCer Tubes: Sar1 was expressed and purified in the same manner as Hariri et al. (2014). Sar1⋯GalCer tubes were produced by incubating GalCer tubes with 1.5 mM GTP (or 5′-Guanylyl imidodiphosphate (GNPPNP)), and 0.7 µM Sar1 for 2 h at 42 °C. Tubes were assessed through negative stain TEM (2% Uranyl acetate) on a CM120 BioTwin and then cryogenically frozen with a FEI Vitrobot on c-flat holey carbon grids (2/2, 300).

Data collection and processing: All data collected in-house was done on a Titan Krios 300 kV with a DE64 detector in integrating mode. Frames were collected in the Appion/Leginon (Lander et al., 2009, Suloway et al., 2005) environment, aligned with MotionCor2 (Zheng et al., 2017), and CTF estimated with both GCTF v1.06 (Zhang, 2016) and CTFFIND v4 (Rohou and Grigorieff, 2015). The final CTF values were chosen based on what had the highest resolution agreement between the estimated and measured CTF. Tubes were manually selected along the long axis of the filaments, though no symmetry was supplied. Particles were roughly aligned during stack creation so that the filament axis aligned with the Y axis. The helical step was small enough to capture each asymmetric unit (ASU) in its own box, as discussed below).

2D classification of Sar1⋯GalCer Tubes: Initial 2D classification of Sar1⋯GalCer tubes was done in cryoSPARC (Punjani et al., 2017). 220,762 particles were classified into 50 classes, of which 7 classes with 64,170 particles were selected as the decorations were visible.

3D refinement and validation: cisTEM (Grant et al., 2018) was used for most of the 3D alignment and refinement. 3D refinement followed a set sequence. 1) Global refinement of theta, phi, and y, 2) local refinement of phi and y, and 3) then local refinement with no restrictions. In general, only one round of each refinement was necessary, but in a few cases more than one round was run. RASTR uses RELION subtraction, so all maps are run through RELION 3D refinement without alignment prior to RASTR processing in order to determine the weighting necessary for RELION reconstruction. The final map was masked to just the upweighted region and sharpened using Phenix Autosharpen Map (Terwilliger et al., 2018). Map validation was done with Phenix Mtriage (Afonine et al., 2018) and ResMap (Kucukelbir et al., 2014).

### 2.2 RASTR methodology

**Fig. 2:**
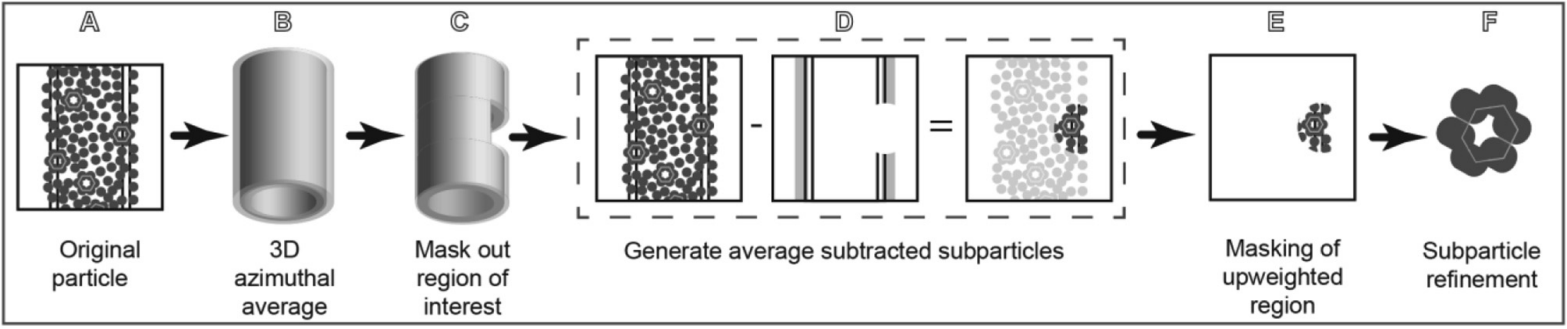
RASTR procedure schematic. A Single particle data. B. An azimuthal-averaged (AA) model is generated from the tubule data. C The region of interest is masked out in the aa model C The masked aa model is projected and subtracted from the original particles, upweighting the region of interest. D The region of interest is masked. E The individual regions are aligned and refined as single particles.

The objective of RASTR is to isolate discrete areas on the surface of the tubule. To properly isolate these surfaces, we generated a methodology which consists of distinct steps that we wrapped in a program called RASTR.py. Here we present the methodology, which is also shown schematically in Fig. 2.

A. During the first step of the RASTR process, tubules are aligned, and their Euler angles randomized about the azimuthal axis which generates an azimuthal-averaged (AA) model (Fig. 2A and B). We typically use cisTEM and an in-house python script to randomize phi for this step due to the ability to control the Euler search parameters with cisTEM, but theoretically any 3D refinement software should suffice. After randomization, the tubules are reconstructed generating an AA model. The input files necessary for RASTR are the original micrographs, AA model, and the phi-randomized star file. The user will decide the center and volume of the region of interest. These values will be dependent on the individual sample, specifically the diameter of the tubule, the size of the asymmetric unit, and whether the sample is a protein filament or sitting on a membrane support. The dimensions chosen should be large enough to encompass at least one complete ASU.

B. RASTR first masks out a region of interest in the AA models then generates *n* models with the masked region at an angle of (360/*n*)° (Fig. 2B). To accomplish this, a mask in created in numpy with the same dimensions as the input model and populated with ones except for a sphere centered at the given *×* value and of radius *r*, populated with zeroes. This array is outputted as a 3D model (the mask) and low-pass filtered to generate a gaussian edge (size of gaussian padding is decided by the user). The mask is multiplied by the input AA model, resulting in a tubule with a sphere masked out (model-phi000.mrc). This is repeated with a new sphere rotated in increments of (360/*n*)°, generating (Model-phi(360/*n*).mrc, Model-phi(2*(360/*n*).mrc, … ,Model-phi((*n*-1)*(360/*n*).mrc) (Fig. 2C). By generating multiple models rotated about the azimuthal axis, we are able to capture the entire circumference of the tubule, with each ASU captured in its entirety, as well as generating overlap for alignment.

C. RASTR generates projections of the masked AA models then subtracts them from the original micrographs (upweighting the regions of interest relative to the background) (Fig. 2D). Importantly this also downweights signal from the opposite side of the tubule which was projected in the same area. Relion_project is used to project and subtract the masked tubules (model-phiXXX.mrc) from the original micrographs. The initial star file for the AA model provides the Euler angles, shifts, and CTF. Projection and subtraction is done for each model, generating an upweighted stack for each.

D. The upweighted regions are then further masked (Fig. 2E), leaving only the upweighted area of interest, allowing isolation of one face. Using the provided Euler angles from the supplied star file, the center of the masked area is tracked from the masked model to the upweighted stack using the rotation matrix below, where Z_1_ is phi, Y_2_ is theta, and Z_3_ is psi. The rotation matrix generates a new (*x*,*y*,*z*) but the *z* is dropped because the model is being projected on a 2D plane. (*x,y*) are offset by the provided *×* and *y* shifts. A new stack is then created masking the pixels outside the upweighted area with the mean value of the original particle image.

$$Z_{1}Y_{2}Z_{3}=\left[\begin{array}{ccc} \cos Z_{1}\cos Y_{2}\cos Z_{3}-\sin Z_{1}\sin Z_{3} & -\cos Z_{3}\sin Z_{1}-\cos Z_{1}\cos Y_{2}\sin Z_{3} & \cos Z_{1}\sin Y_{2}\\ \cos Z_{1}\sin Z_{3}+\cos Y_{2}\cos Z_{3}\sin Z_{1} & \cos Z_{1}\cos Z_{3}-\cos Y_{2}\sin Z_{1}\sin Z_{3} & \sin Z_{1}\sin Y_{2}\\ -\cos Z_{3}\sin Y_{2} & \sin Y_{2}\sin Z_{3} & \cos Y_{2} \end{array}\right]$$

The AA model is a smoothed estimate of the face, so while the overall intensity of the downweighted part of the signal is reduced, the some signal will remain (Fig. 2D, right panel). Thus, when the subparticle is reconstructed, the residual signal will generate some noise in the final model in the region outside of the upweighted region. In other words, since the signal subtraction is not perfect, the residual density outside of the upweighted region will always be present in the reconstruction. This does not appear to affect resolution as it generates a degraded structure outside the upweighted region which can be masked out as discussed in Results and Discussion. If desired, the area of upweighting can be extracted into a smaller box, though this process is optional, and we have found it increases the noise in the final model. For RASTR arguments see Supplemental Section 1.

E. In the last step, the star files of the masked particles are concatenated into a single star file (directing to the various stacks). Relion_preprocess is run to create a single stack and star file and relion_reconstruct is used to generate an initial model for alignment and to spot-check proper upweighting/masking and alignment. After RASTR processing, the RASTR particles are treated as a stack of single particles. Since the particles are removed from the surface of a tube they are limited in their degrees of freedom for alignment and this can be used to eliminate poorly aligning particles in the refinement. If the psi and theta alignment were perfect before RASTR processing, then only phi and y-shift would need to be refined. This is obviously not the case, but psi, theta, and x-shifts should be limited to small changes, and any large deviations can be dismissed as misalignment.

## 3 Results and discussion

### 3.1 Bare GalCer tubules

**Fig. 3:**
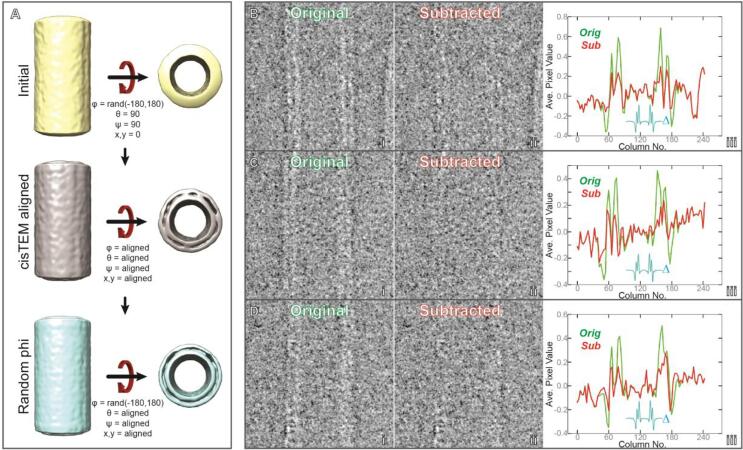
Subtraction of bare GalCer tubules. A GalCer tubes were roughly aligned vertically in two dimensions during stack creation then φ was randomized between −180° and 180°; and θ,ψ were set to 90° for reconstruction (top). All Eulers and shifts were aligned in cisTEM (middle). φ was once more randomized between −180° and 180°, creating an azimuthal-averaged model (bottom). B–D Examples of undecorated GalCer tubule particles before (i) and after (ii) subtraction with relion_project. (iii) The average pixel value of each column (excluding a ten pixel border along the edges of the image). Original image (green) and subtracted (red), with the difference inlayed in cyan (Δ).

The success of RASTR is highly dependent on the effectiveness of the model subtraction. To test subtraction, we collected cryo-EM images of undecorated GalCer tubes (See Supp. Table 1 for data collection statistics), aligned in cisTEM (Fig. 3A) and an AA model was generated (Fig. 3A, bottom). The AA model was subtracted from the original particles (Fig. 3B-D), in this case without specifying a region for upweighting so that the complete tubule could be subtracted.

The azimuthal-averaged model was subtracted from the original particles using relion_subtract. Three representative samples are shown in Fig. 3D-F. Each particle has features that illustrate the pros and cons of our current method of subtraction. Particle B was generally straight with a distinct bilayer, but had a slight deviation in diameter and a kink at the top. Particle C was an ideal particle, as it was straight with a distinct bilayer. Particle D was straight, but the right bilayer was not distinct. The average pixel value of each row (with a ten pixel border to remove artifacts from normalization) is presented in Fig. 3B–D(iii) to provide an objective measure of the subtraction. The subtraction worked well for all cases but was most successful on particle C, with the average values almost reduced to noise, while remnants can be seen in both particles B and D. The most consistent subtraction was the center sections of the particles, away from the edges. It should be noted that the diameters of the tubes have the potential to have a large effect on the subtraction. If the particles’ diameters deviate from the AA model, then instead of subtracting features the subtraction will create artifactual negative densities. For particles with a consistent diameter (including most tubule filaments), one model suffices, but for tubules that vary in diameter, the particles would need to be classified before alignment and distinct azimuthal-averaged models generated for each class. Subtraction near the edges of the box created artifacts. The artifacts are likely caused by the normalization algorithm commonly applied to initial stacks. To provide the combination of proper coverage of each ASU and avoiding the borders means that the overlap along the azimuthal axis should be generated during initial stack creation. Overlap should be enough so that each ASU will be captured in a separate box.

### 3.2 Ideal VipA/VipB

**Fig. 4:**
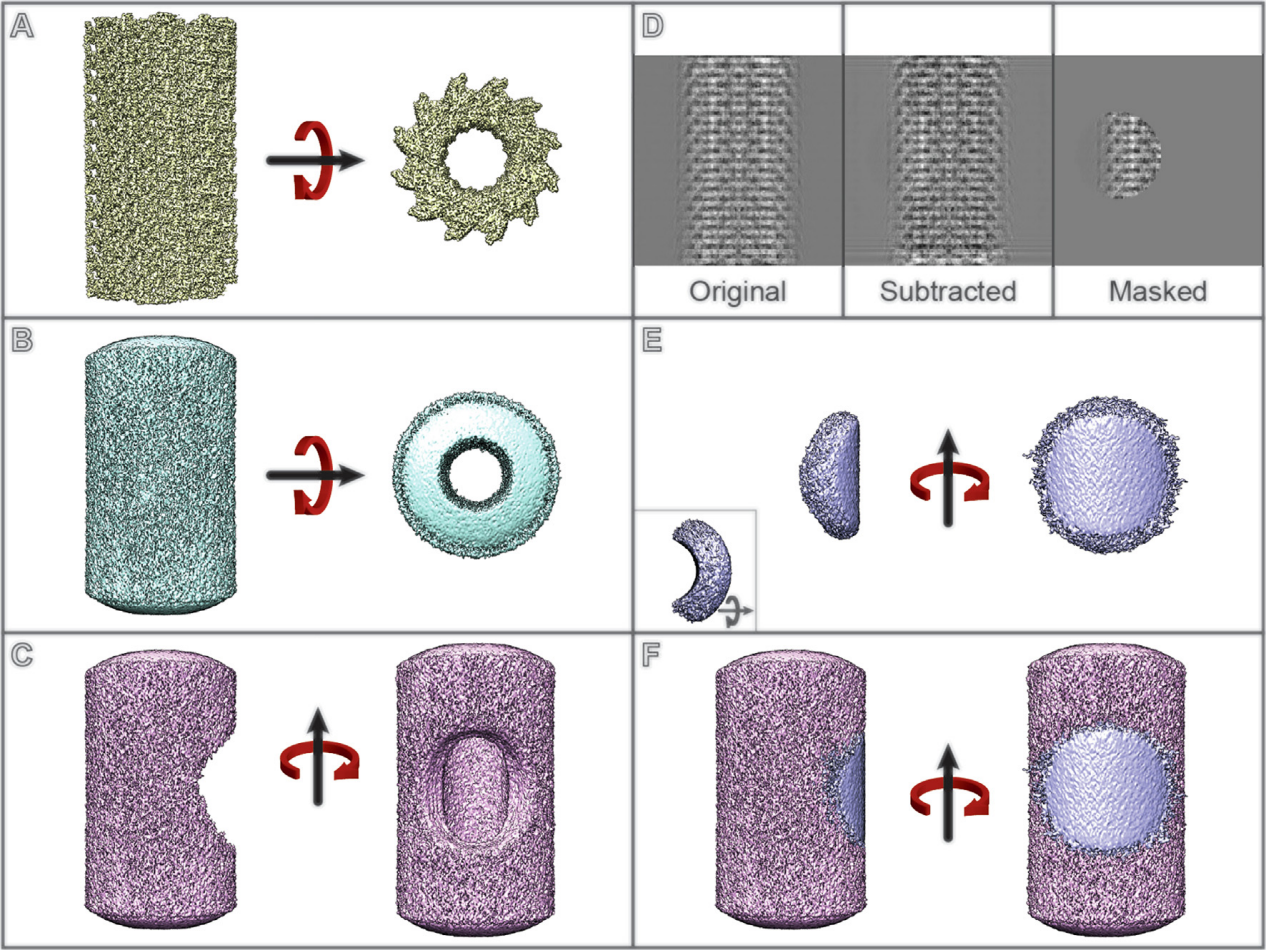
RASTR summary using ideal VipA/VipB data. A Extended VipA/VipB model generated from pdb 3J9G. B Azimuthal-averaged VipA/VipB model. C Model to be subtracted from the particles with the section to upweighted removed. D Particle progression through RASTR, from left-to-right: original particle, subtracted, and then masked. E Reconstruction of the final RASTR particles using the Eulers and translational shifts from the azimuthal-averaged model. F Overlay of the model to be subtracted (C) and reconstruction of the RASTR particles (E).

**Fig. 5:**
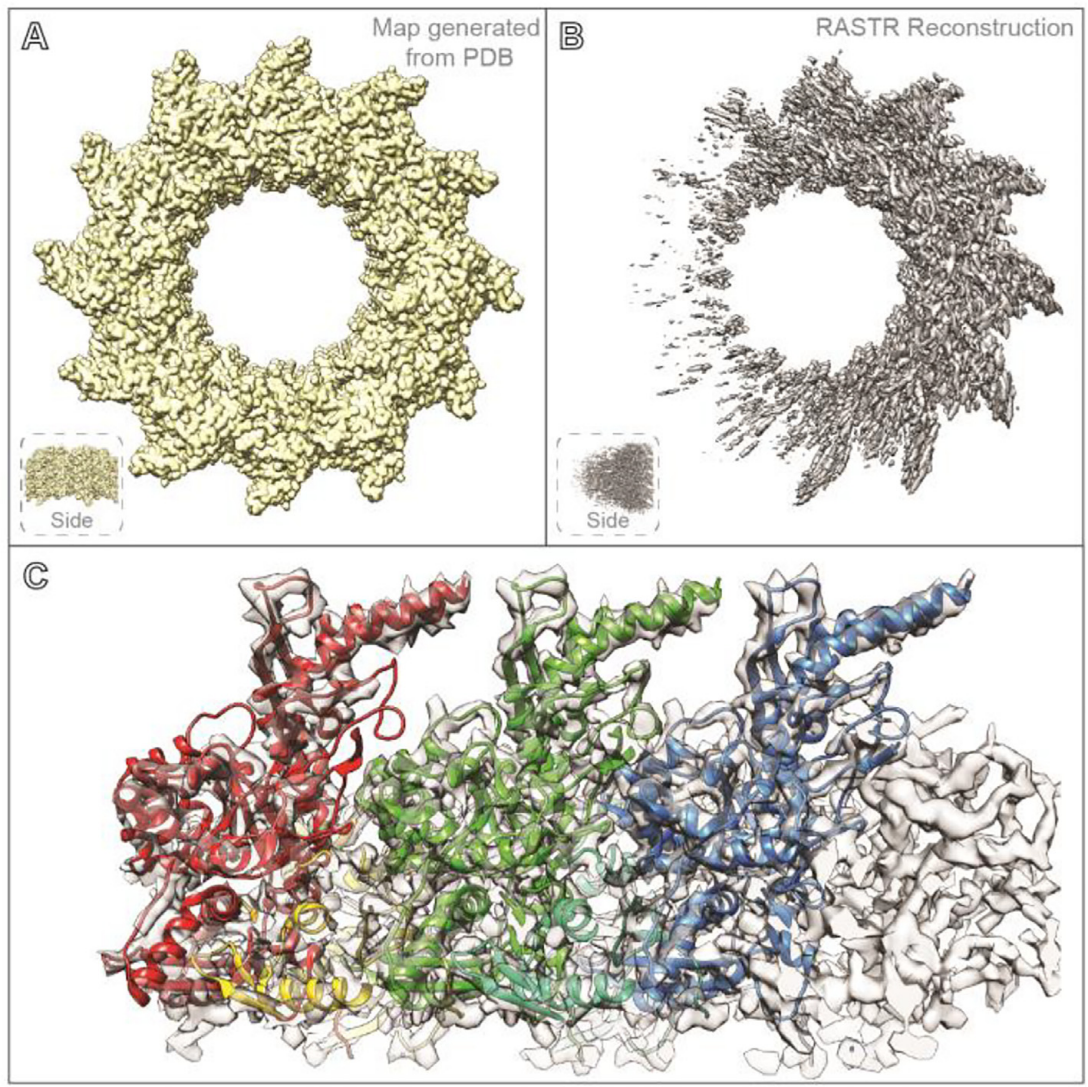
Ideal data (generated VipA/VipB) filament alignment. A Original VipA/VipB pdb model (3J9G), extended along the azimuth. B Aligned map of RASTR VipA/VipB particles. The middle right section of the aligned map resolved to a high resolution, which degrades as the angle away from the middle right increases. C Overlay of (A) and (B). D Middle right section of the aligned map fit the original data, showing secondary structure.

After the individual sections of RASTR were tested, we tested RASTR on simulated helical data in order to assess the ability of the approach to faithfully reconstruct a biological filament. Ideal simulated images of tubular filaments were created using the VipA/VipB pdb model (Kudryashev et al., 2015) (see Methods), with the original particle stack containing 3550 particles, equivalent to 426,000 ASUs (Fig. 4D, left). Reconstruction of the projected particles regenerated the original ideal map (Fig. 4A). An azimuthal-averaged model was created (Fig, 4B) and processed in RASTR, upweighting a radius 50 spherical section, 50 pixels from the center of the box on the *x*-axis and repeated at increments of 15° (360°/24) around the azimuth (Fig. 4C). The final stack contained 85,200 sub-particles (Fig. 4D, right), equivalent to 150,000 unique ASUs. Particles were reconstructed using the azimuthal-averaged Eulers and shifts (offset by 15° increments) (Fig. 4E), which recaptured the missing area from the original subtraction model (Fig. 4F). Using cisTEM, we were able to successfully align the ideal RASTR VipA/VipB data, resolving the original structure only in the region we had upweighted (Fig. 5). Since there was residual density in the images due to the imperfect subtraction of the AA model, there was elongated and diffuse density in the 3D reconstruction in the regions outside of the upweighted region of the tubule. (Fig. 5B). Local resolution calculated in ResMap confirmed this result, with the upweighted region showing a resolution around 4 Å (Nyquist), which rapidly deteriorates as one goes farther from the region until all density is lost (Supp. Fig. 2,3). The RASTR aligned map recaptures the details of the original model, from the core filaments to the extended α-helices and loops (Fig. 5C). The reconstruction of the ideal data from VipA/VipB provided a blueprint for how to treat filaments in RASTR. The lack of definition in the areas outside the upweighted section, meant a mask was necessary or the reconstruction began to degrade. Another issue we found was during initial alignment the particles tended to bunch around the starting phi angles producing an extremely low-resolution map. Allowing the other angles freedom to align only exacerbated the issue as the particles were still bunched but they would diverge from reasonably allowed translational and rotational alignments for the tube resulting in a junk reconstruction. Fixing the alignment to just phi and y-shifts until the particles spread out around the azimuthal axis and had an initial alignment then freeing the other angles to refine resulted in a significantly better reconstruction (See Euler angle distribution Supp. Fig. 3).

We note, however, that we were unable to converge on the correct structure without having a good starting model. We expect this was due to inability to separate out the polarity of the helical tubule during refinement. Low resolution starting models were suitable to resolve the high resolution structure as long as they had sufficient features to determine polarity. We successfully refined maps to Nyquist with a 20 Å model but failed with a 30 Å model (Supp. Fig. 1). Two exposed helices (VipA H4 and VipB H1) on the outside of the VipA/VipB filament produce a low resolution hook feature which allows for orientation in theta/psi, while in the lower resolution (30 Å) model this feature appears as just a point, with no directional value (Supp. Fig. 2). When the particles were tracked and the proper polarity known and accounted for in the initial star file, the 30 Å model was also able to produce a similar high resolution model. We expect that a more sophisticated Euler search could overcome this problem. Due to the production of the azimuthal average, the Eulers for the subparticles generated by RASTR should be fairly close to their optimal angles, so searching angles of plus or minus 90° for theta and a narrow range of phi should be sufficient for a high quality reconstruction (see Supp. Fig. 3).

### 3.3 Experimental VipA/VipB

Given the success with RASTR on simulated data, we applied the technique to real experimental cryo-EM data. Experimental VipA/VipB cryo-EM data (Kudryashev et al., 2015) are available on the EMPAIR database, and these were obtained from EMPIAR and processed in RASTR. The VipA/VipB structure was originally resolved using Iterative Helical Real Space Reconstruction (IHRSR) to a resolution of 3.5 Å (Kudryashev et al., 2015). For RASTR, the filaments were manually picked, and 480x480 pixel stacks were created with 1 Å/pixel. The helical step during stack creation (step along the azimuthal average to generate the next box) was 50 Å, generating significant overlap between particles, which was necessary to maximize the averaging of asymmetric units during RASTR processing. The VipA/VipB filaments were processed in RASTR, upweighting a 100 pixel radius spherical section that was centered on the highest density of VipA/VipB (100 pixels from the azimuthal axis). cisTEM was used to align the RASTR processed VipA/VipB data resulting in a final map with a reported resolution of 4 Å which is lower than the reported IHRSR structure, however the reconstruction of the RASTR processed particles produced a map with comparable features to the original helically aligned one.

**Fig. 6:**
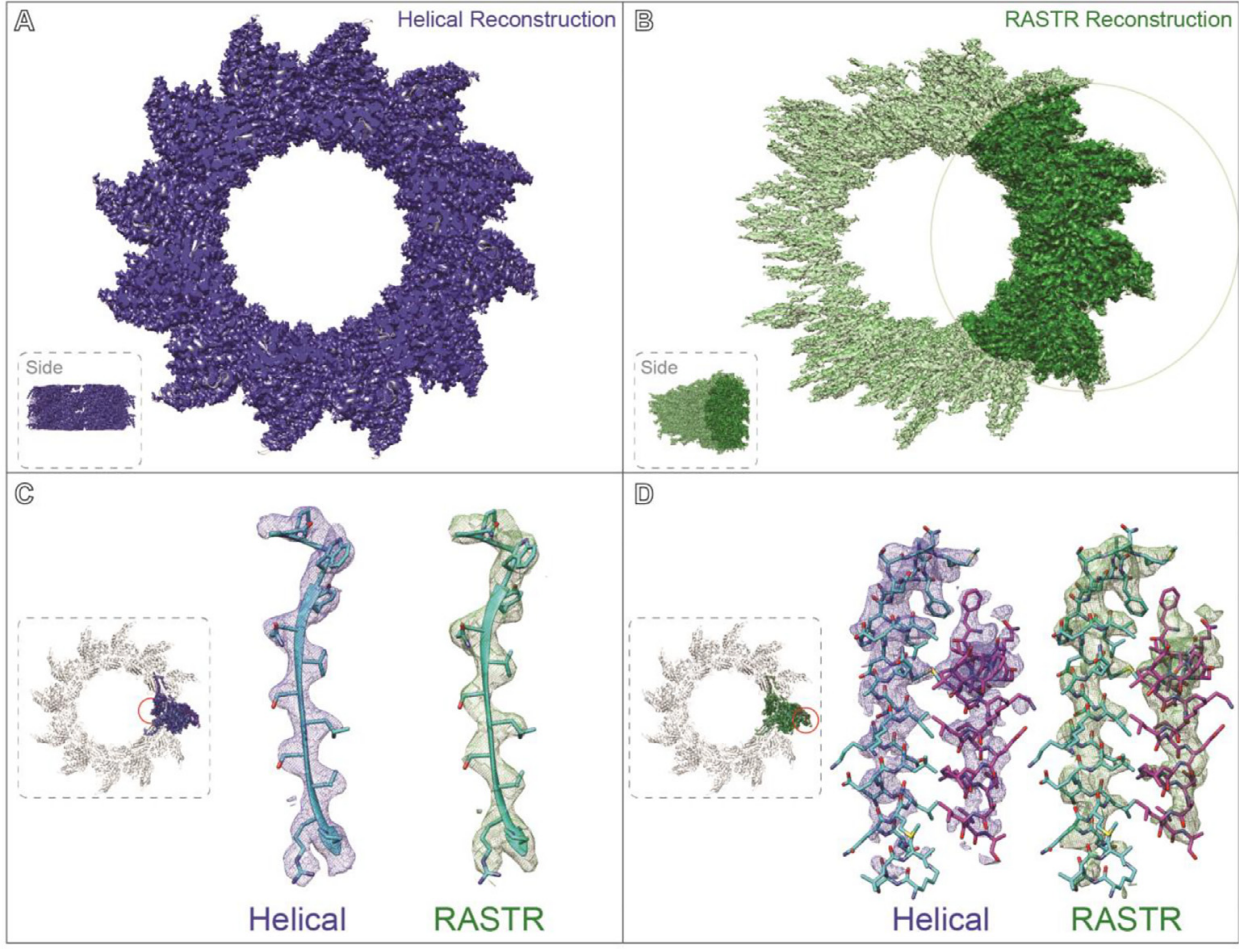
Experimental VipA/VipB sheath reconstruction. A Original reconstructed map aligned using IHRSR (blue). B RASTR processed reconstruction with upweighted area highlighted (green). C Domain 1 β-sheets from the inner surface. Both maps had good detail, with the original model generated from the IHRSR alignment fit in the new RASTR map. D Two outer surface α-helices from Domain 3 had similar coverage and resolution in the IHRSR map (blue) as the RASTR map (green).

**Fig. 7:**
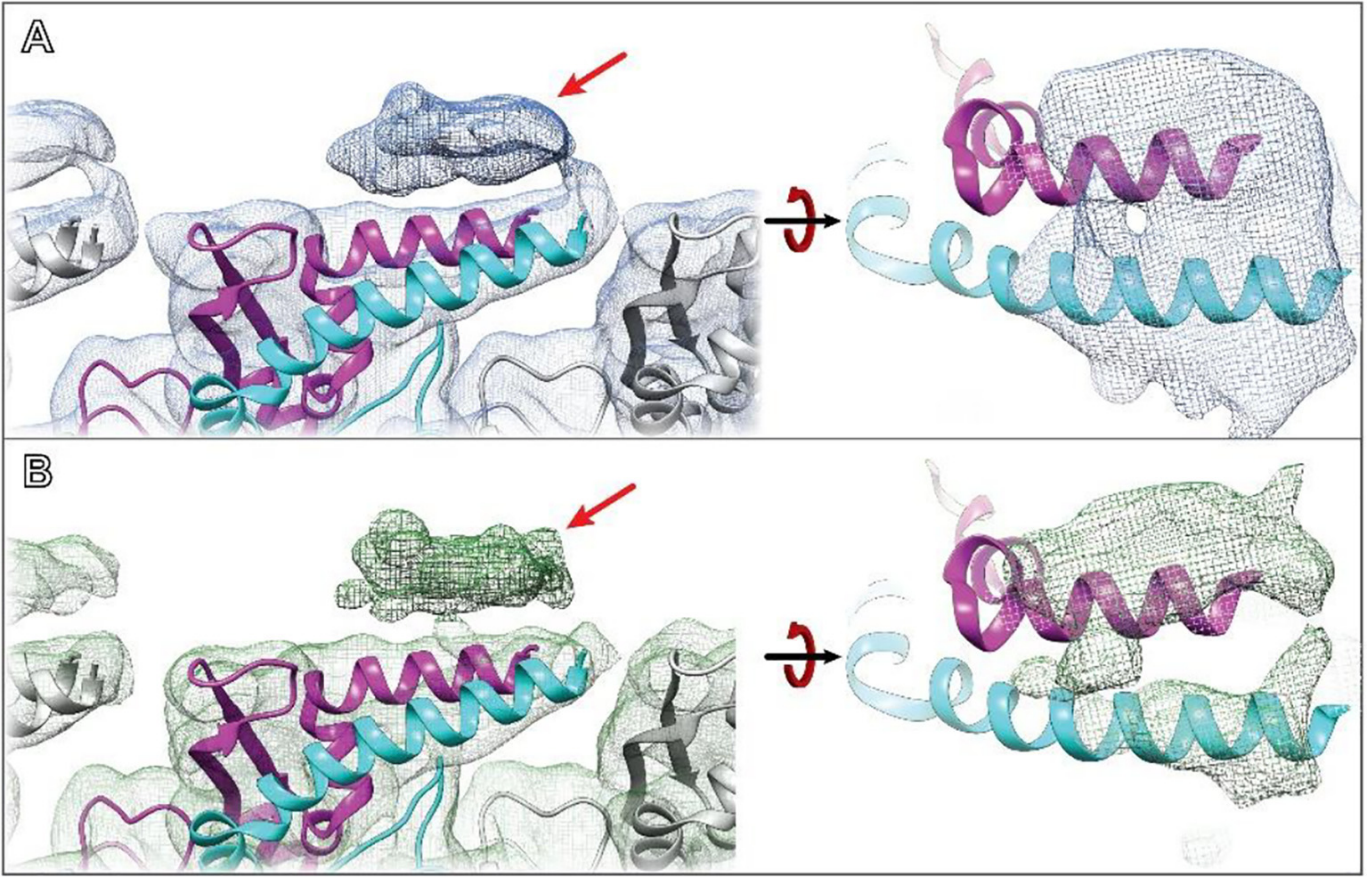
VipA/VipB sheath unmodeled density. A Original map filtered to 7.5 Å highlighting the unmodeled density identified after alignment (left) with top view (right). B RASTR map filtered to 7.5 Å exhibiting the same density (left) with top view showing two distinct oblong densities running parallel, map is removed from helices for clarity.

As with the ideal data, RASTR processed data produced a high-resolution map in the upweighted sphere but with deteriorating quality outside of the upweighted sphere (Fig. 6B). Since the only valid part of the 3D map was inside the upweighted region, all subsequent analysis was limited to that section (Fig. 6B, highlighted). The threshold was normalized between the original IHRSR map and the RASTR reconstructed map by zoning the maps around a single VipA/VipB unit (one VipA chain and one VipB chain) and setting the threshold so the enclosed volumes were equal. Both maps produced a high enough level of detail, including side chain density, for the architecture from the inner surface (Fig. 6C) through the buried residues (referred to as Domain 1 and Domain 2). Two exposed α-helices (VipA H4 and VipB H1) on the outer surface of the filament (Domain 3), showed overall less density in both maps (Fig. 6D), but were comparable between the IHRSR map and the RASTR one. In addition, we were able to recapture a section of unmodeled density that was observed in the original helical reconstruction (Fig. 7, maps filtered to 7.5 Å). This density had higher occupancy for the RASTR map and resolved as two distinct elongated densities running parallel to each other (Fig. 7, right panels).

Despite the success in reconstructing the VipA/VipB structure using RASTR, estimating the resolution by FSC proved to be unreliable due to the masking imposed during isolation of the RASTR subparticles. FSC calculated from half-maps did not approach zero, so could not be used for reliable resolution estimation (Supp. Fig. 4A, 4B). As an alternative, we used RESMAP (Supp Fig. 5,6) which reported local resolution from 3.2 to 4.2 Å. Additionally, we used map-model FSC to try and estimate the overall resolution. In the case of the VipA/VipB tubules, the map-model FSC of the upweighted sphere at 0.5 was 4.17 Å (Supp. Fig. 4C), when masked to a single VipA/VipB pair near the center of our upweighted region the FSC at 0.5 was 4.07 Å (Supp. Fig. 4D). While our reported resolution does not approach the original 3.5 Å map, we believed based on the features seen in Fig. 6, Fig. 7 that within the upweighted region the map was of similar resolution.

Processing of the VipA/VipB sheath using the RASTR method demonstrated that it can be used to generate 3D reconstructions of tubular particles with comparable quality than helical reconstruction without imposing helical symmetry.

### 3.4 Sar1⋯GalCer tubules

**Fig. 8:**
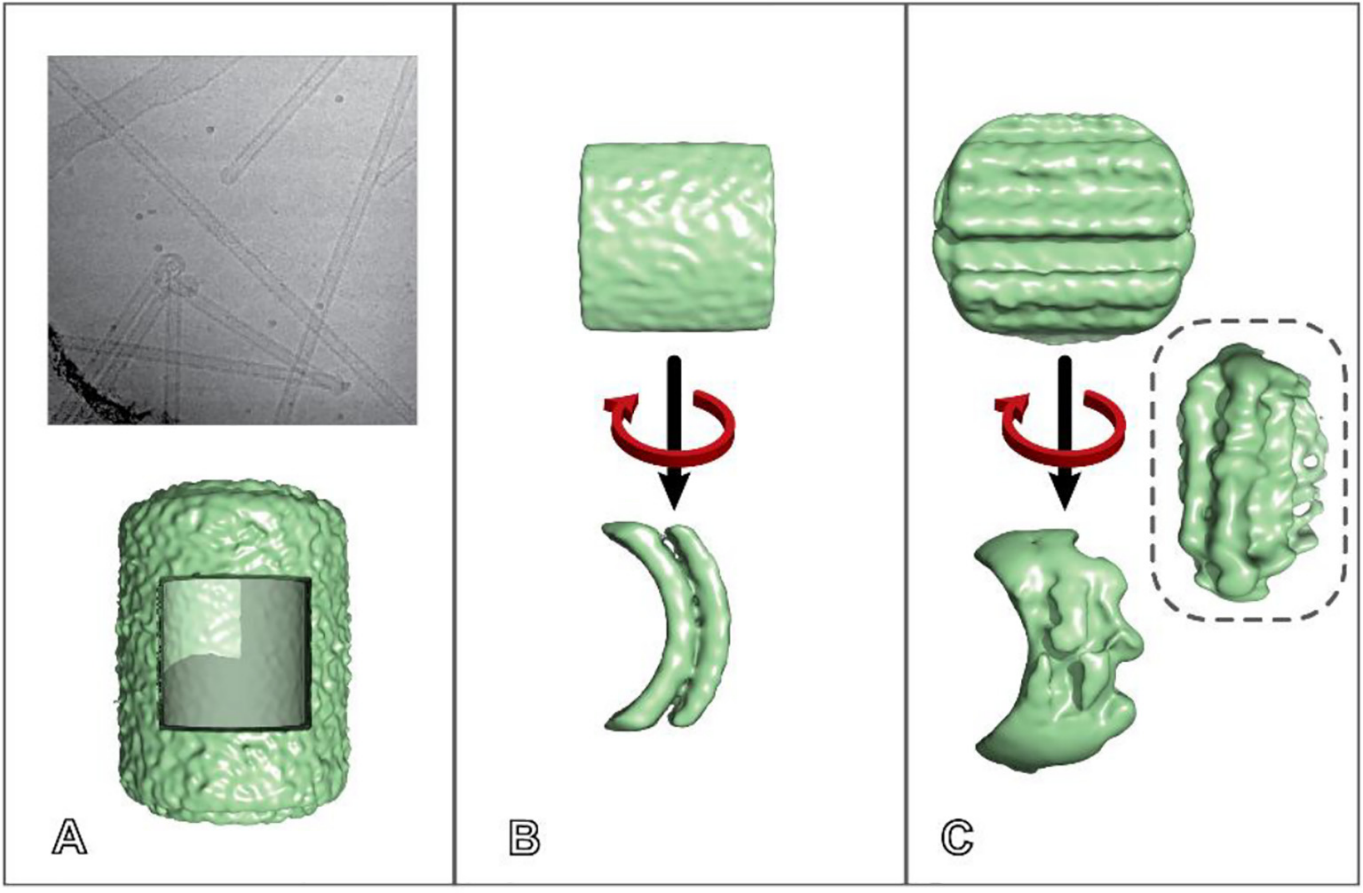
Sar1 tubules data collection and alignment. A Sar1 tubule micrograph (top) with azimuthal aligned average with subsection to be aligned (bottom). B After RASTR the subsection reconstructed using the azimuthal averaged Euler angles. C After alignment and classification a pattern of linear lines appears on the outside of the tubules, which gains definition during masking, the dashed structure is after free refinement in cisTEM.

Given our success using RASTR on a helical tubule, we tried the technique on Sar1 decorated membrane tubules that have previously been shown to have local order but no long range symmetry. Previously our group showed that Sar1 oligomerizes on GalCer lipid tubules but could not be reconstructed to high resolution due to the lack of coherent long-range symmetry when it oligomerizes on membrane. We used RASTR to characterize the way that Sar1 oligomerizes on membrane. We collected cryo-EM data of Sar1 coated membrane tubules (Supp. Table 2). Tubes were then segmented aligned and classified with 2D classification on whole segments. 2D classification successfully separated bare tubules and decorated tubules. (Supp. Fig. 9). As with our previous study, Sar1 could be observed to bind the tubules, but no long-range order was observed. We next subjected the tubules to RASTR processing (Fig. 8A,B). In this way, we were able to separate membrane surfaces with bare membrane from ones with Sar1 bound. Once we knew a tubule was bare, we could discard all the particles from that specific tubule, generating a stack of decorated tubules only. Within the decorated tubules, we were able to use 3D classification to separate out ordered decorations from disordered decorations. This revealed that Sar1 oligomerized as long strings of protein that run along the flat tubular axis, with only very weak lateral interactions (Fig. 8C). The lack of lateral association was likely due to the radius of the tubules. Our previous studies showed that Sar1 made 2D lattices on relatively flat membrane but the order broke down on tubules with diameters lower than 100 nm (Hariri et al., 2014). Our new results using RASTR reveal that the reason for this was that the increased curvature of the GalCer tubes caused the lateral interactions between Sar1 to break, leaving only the vertical interactions. These observations would not have been possible without the use of RASTR.

## 4 Conclusions

RASTR provides a new tool for processing tubules without applying symmetry, making it more flexible than current processing methods. We anticipate that this approach will enable structure determination for a variety of biological molecules that were previously inaccessible. We expect that this approach will be particularly powerful helical tubules with non-helical decorations and tubules with limited symmetry. Filament decorations can be examined in their natural state, bound to filaments, and their structures and binding interfaces can be explored, while limited symmetry complexes on tubules will be able to be resolved without extensive manual curation and masking. RASTR could also assist in solving the structures of tubules for which the helical symmetry is difficult to determine. While current results provide compelling evidence of the value of the technique, there are many improvements to RASTR that can be implemented. For instance, alignment and classification of RASTR particles could be improved by better subtraction that better accounts for deviation from the model and weights the model better for a cleaner subtraction. Additionally, resolution estimation requires estimating local resolution using map features instead of the typical FSC measurements. Nonetheless, RASTR has the potential to allow processing of a variety of particles that were previously inaccessible except through extensive manual masking and to allow tubular filaments to be solved without needing any knowledge of the symmetry.

## CRediT authorship contribution statement

**Peter S. Randolph:** Methodology, Software, Validation, Formal analysis, Investigation, Writing - original draft, Visualization. **Scott M. Stagg:** Conceptualization, Methodology, Validation, Resources, Writing - review & editing, Supervision, Funding acquisition.

## Declaration of Competing Interest

The authors declare that they have no known competing financial interests or personal relationships that could have appeared to influence the work reported in this paper.
