# Supplementary material for: Reconstruction of Average Subtracted Tubular Regions (RASTR) enables structure determination of tubular filaments by cryo-EM
Source: J Struct Biol X. 2020 Mar 9;4:100023. doi: 10.1016/j.yjsbx.2020.100023 (PMC7337063; doi:10.1016/j.yjsbx.2020.100023)
Supplement: Supplementary data 1 [file mmc1.docx]

**Supplemental**

**Section 1 – RASTR Arguments**

- **RASTR arguments:**

-h, --help Show the help message and exit.

-s, --star_in Initial star file for the azimuthal-averaged aligned model.

-m, --model Azimuthal-averaged model to subtract.

-a, --angpix Angstroms per pixel.

-r, --radius Size of sphere to mask/upweight, in pixels, default is 3/16 of box size

-x, --x_start Center of sphere to mask in x at phi=0, in pixels, IMOD coordinates, default is 3/4 of box size.

-n, --n_spheres Number of models to generate to upweight around axis, convenient if 360 is divisible by n_spheres, default is 9.

-t, --tube_radius Radius of the membrane to be subtracted. This will remove the upweighting of the tubule out to a certain radius. Useful for examining decorations rather than the entire tube.

-c, --center Center the upweighted area in a new smaller box.

-o, --output Output rootname for star/mrcs, default is RASTR_particles.

-k, --keep_scratch Keep scratch folder, default is False with only final star, mrcs, and reconstruction output. Can generate a large amount of data if marked true

-b, --both End with two stacks, one with section masked keeping same box size as original and the second with it centered in a smaller box

-g, --gauss Sets the gaussian edge for the masking of the AA model

**Section 2 – Data Collection Statistics**

Supp. Table 1. Collection Statistics for undecorated GalCer tubes

| Magnification | 29000 |
| --- | --- |
| Pixelsize | 1.29 Å |
| Frames | 22 |
| Framerate | 31.25 |
| Total dose | 61.32 e-/Å^2^ |

Supp. Table 2. Collection Statistics for Sar1∙∙∙GalCer tubes

| Magnification | 37000 |
| --- | --- |
| Pixelsize | 1.01 Å |
| Frames | 54 |
| Framerate | 31.25 |
| Total dose | 60.21 e-/Å^2^ |

**Section 3 – Supplemental Figures**

**Supp Fig 1**


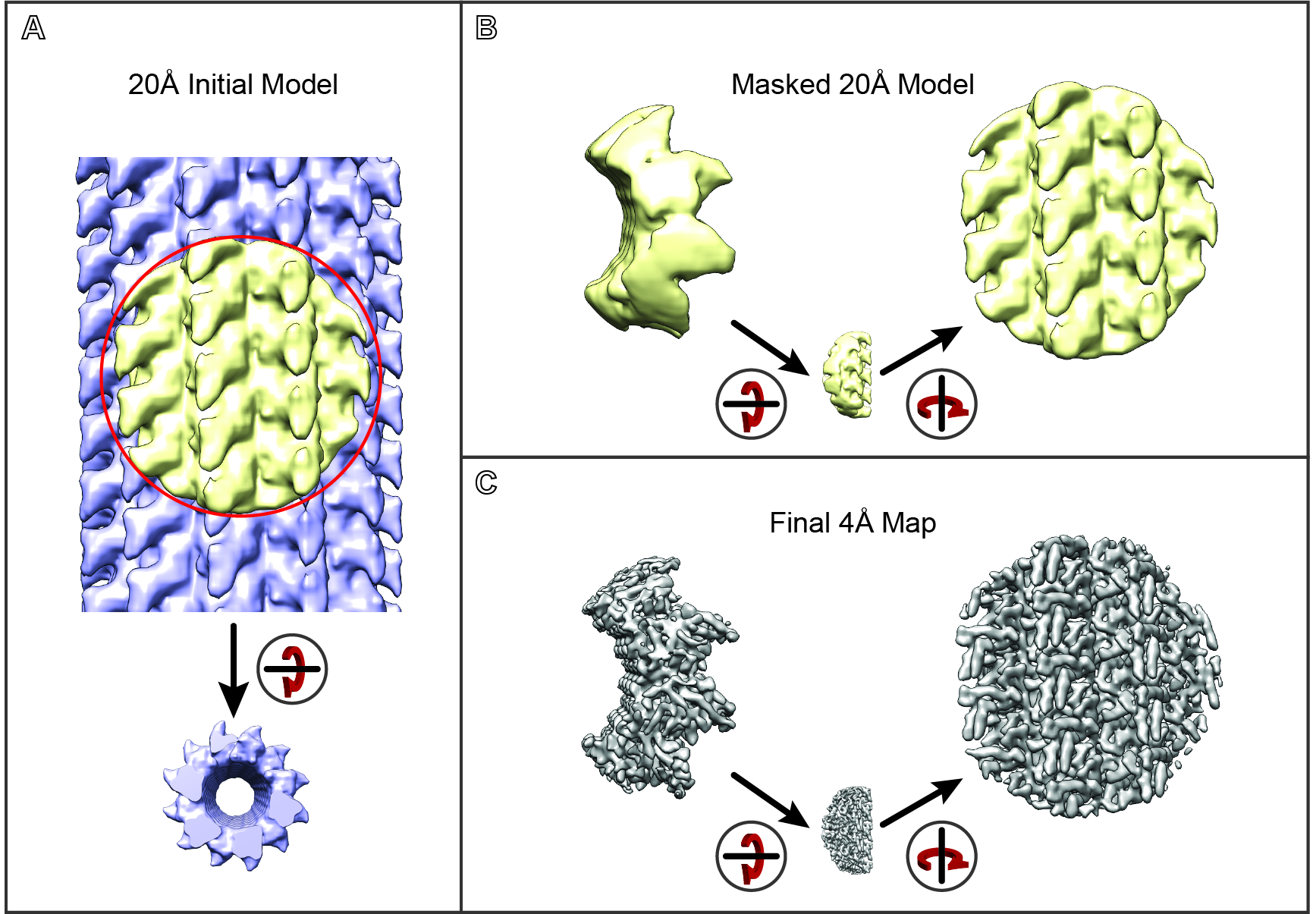


S Figure 1. Model requirements for refinement. A. Initial 20Å model (blue), generated with MolMap from starting data. Model is masked (red circle) to the RASTR sphere generating masked model (yellow) B. Starting model for refinement. C. Final 4Å (Nyquist) model after refinement of RASTR data using initial model (B).

**Supp Fig 2**


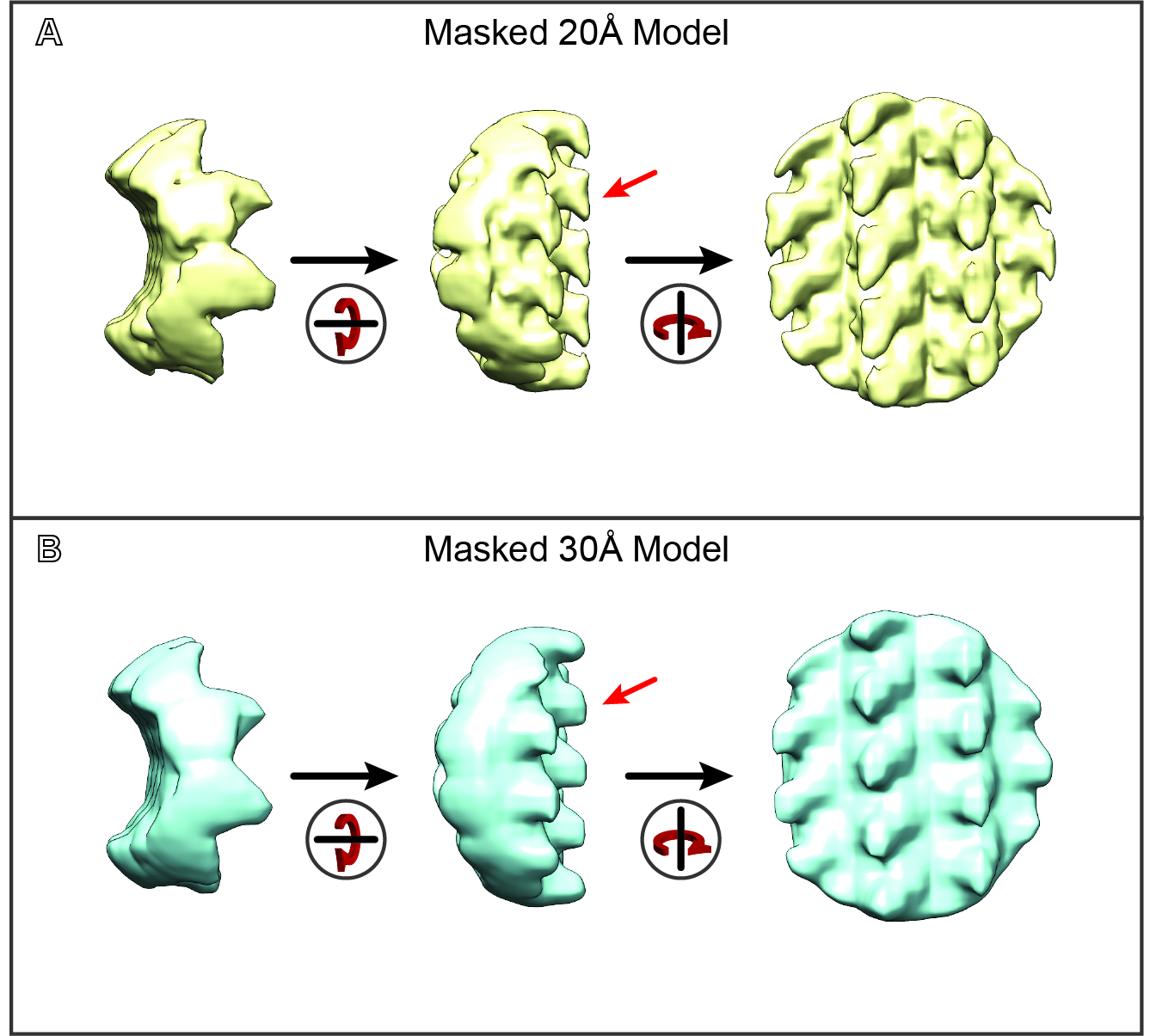


S Figure 2. Starting model comparison. A. 20Å starting model which successfully solved the structure to Nyquist. B. 30Å starting model which unsuccessfully solved the VipA/VipB structure. Lack of detail deters determination of polarity (theta or psi Eulers), leading to a poor resulting model.

**Supp. Fig 3**


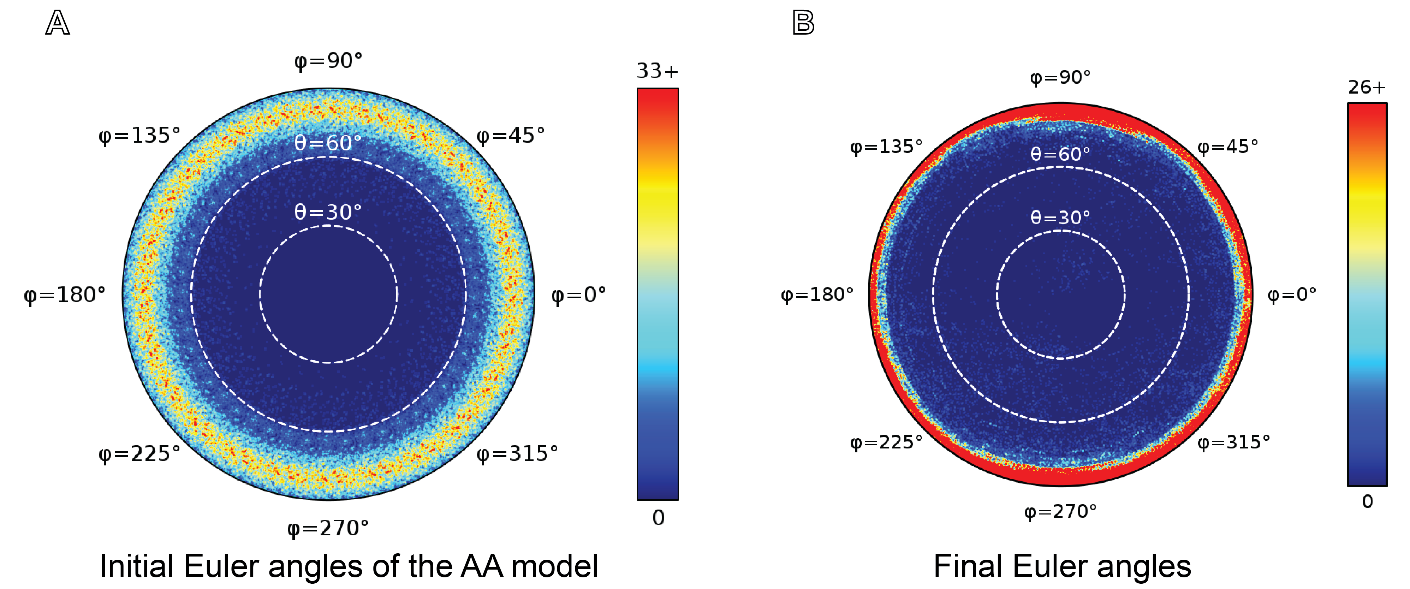


S Figure 3. VipA/VipB Euler Angles. A Initial randomized Eulers for the AA model shows an even distribution. B Final Euler angles demonstrate a similar distribution of phi.

**Supp. Fig. 4**


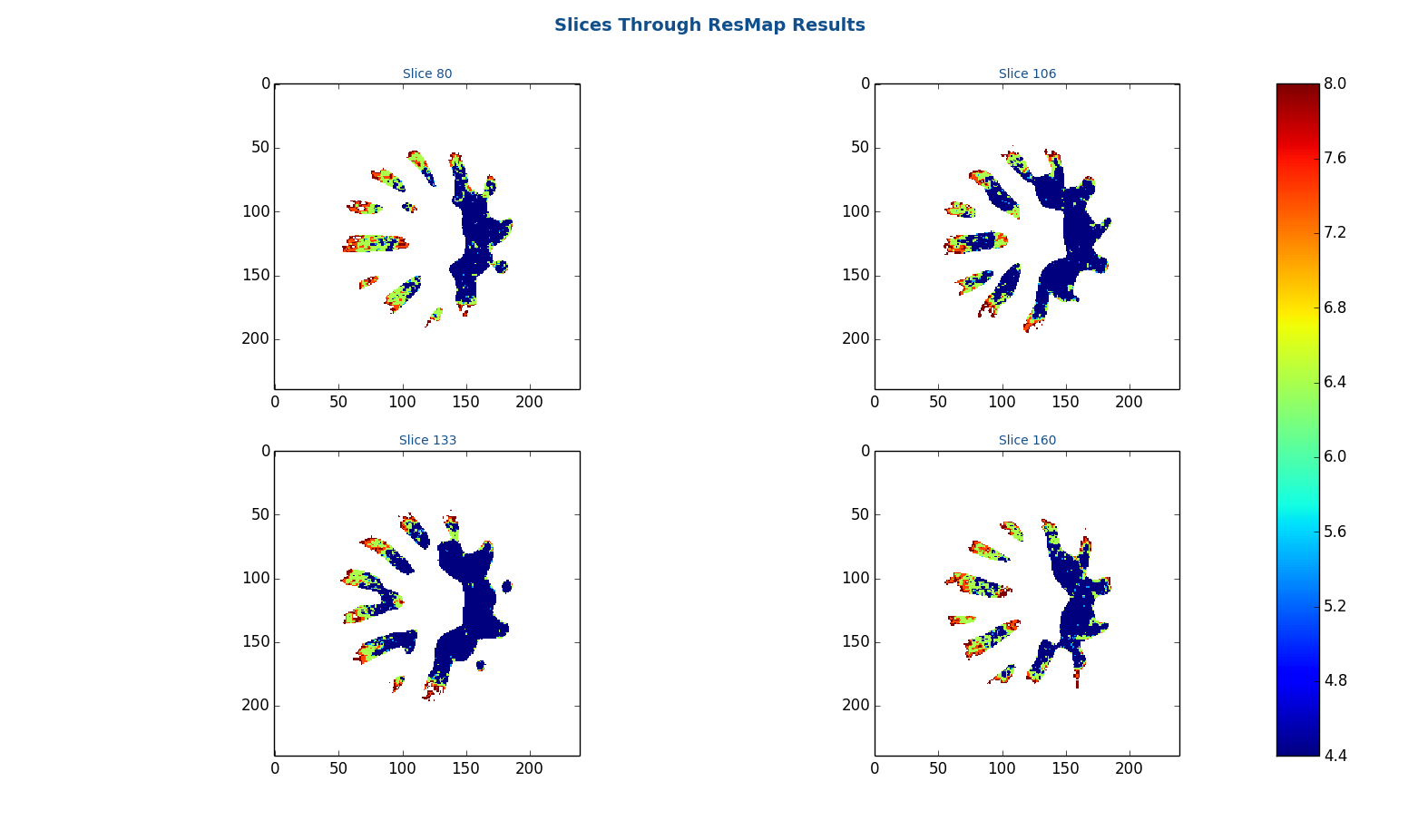


S. Figure 4. ResMap local resolution slices for ideal VipA/VipB. Within our final map (pre-sharpening) local resolution was reported between 4.4 and 8.4 Å.

**Supp. Fig. 5**


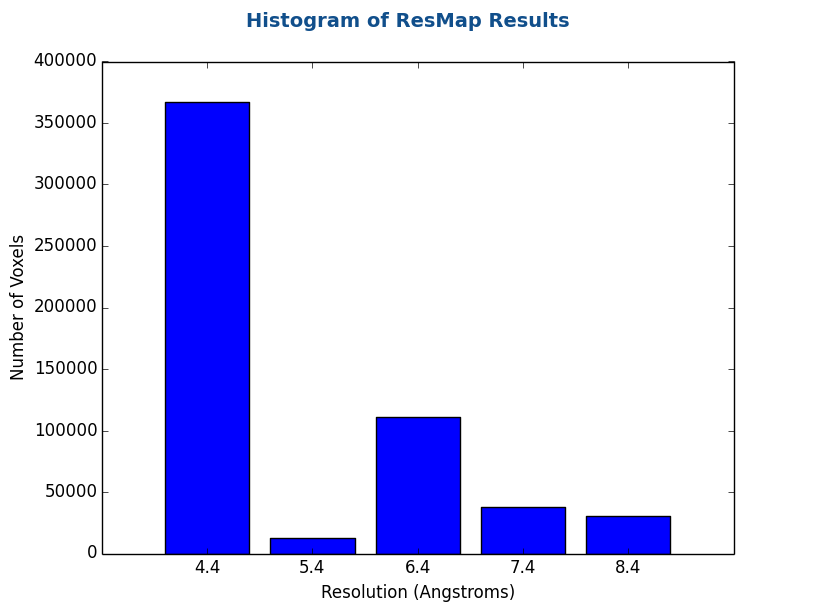


S. Figure 5. ResMap local resolution histogram of ideal VipA/VipB. Within our final map (pre-sharpening) local resolution was reported between 4.4 and 8.4 Å.

**Supp. Fig. 6**


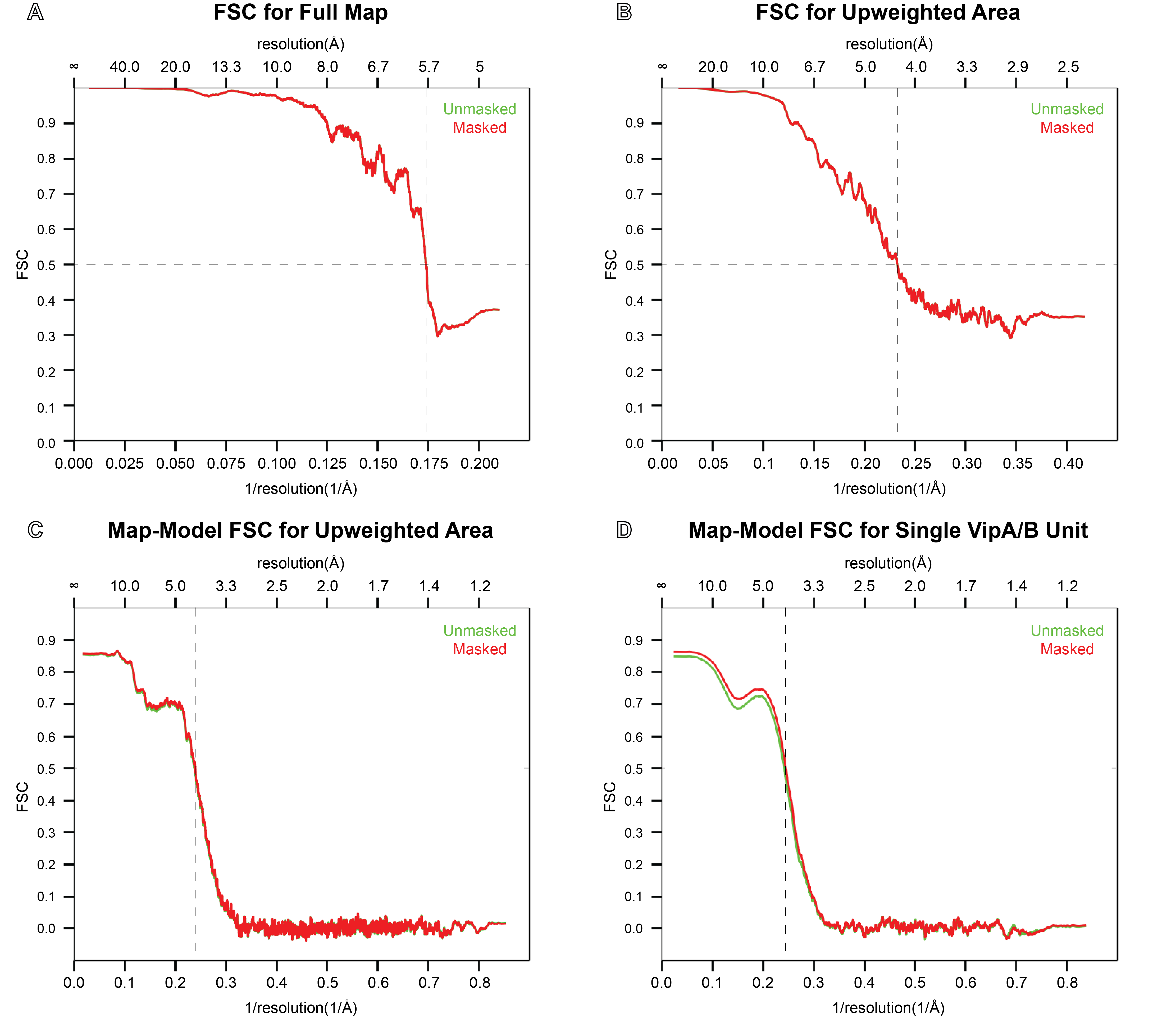


S. Figure 6. FSC calculations for RASTR processed experimental VipA/VipB experimental structure. Calculated in Phenix Mtriage. A Half-map FSC on the VipA/VipB full map including downweighted area (0.5 Res = 5.7 Å). B Half-map FSC of the VipA/VipB map clipped to RASTR upweighted section (0.5 Res = 4.3 Å). C Map-Model FSC on the VipA/VipB map clipped to upweighted section (0.5 Res = 4.17 Å). D Map-Model FSC on a single VipA/VipB subunit (0.5 Res = 4.07 Å).

**Supp. Fig. 7**


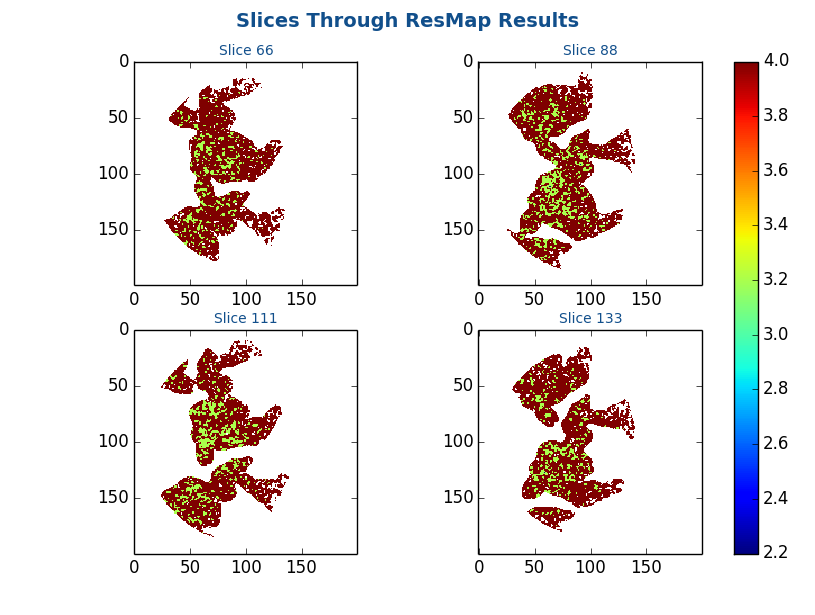


S. Figure 7. ResMap local resolution slices of experimental VipA/VipB. Within our final map (pre-sharpening) local resolution was reported between 3.2 and 4.2 Å.

**Supp. Fig. 8**


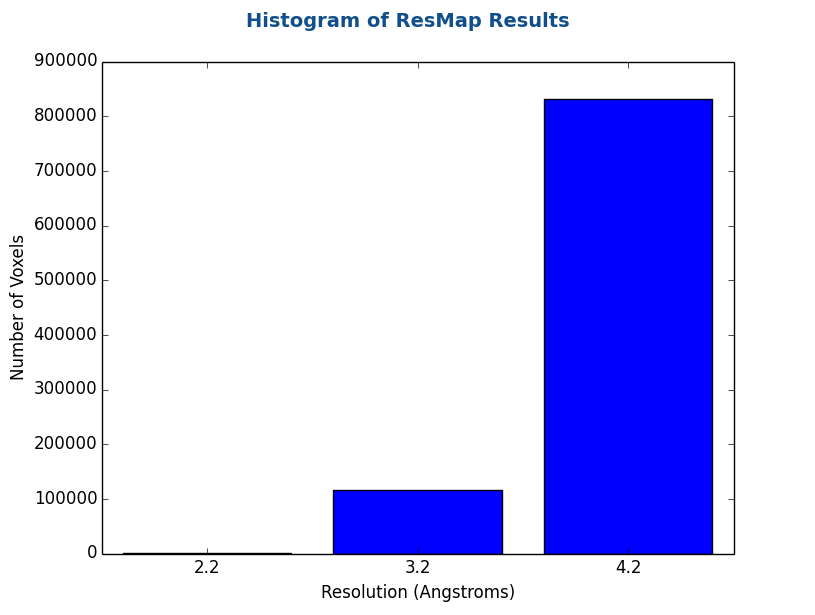


S. Figure 8. ResMap local resolution histogram of experimental VipA/VipB. Within our final map (pre-sharpening) local resolution was reported between 3.2 and 4.2 Å.

**Supp. Fig 9**


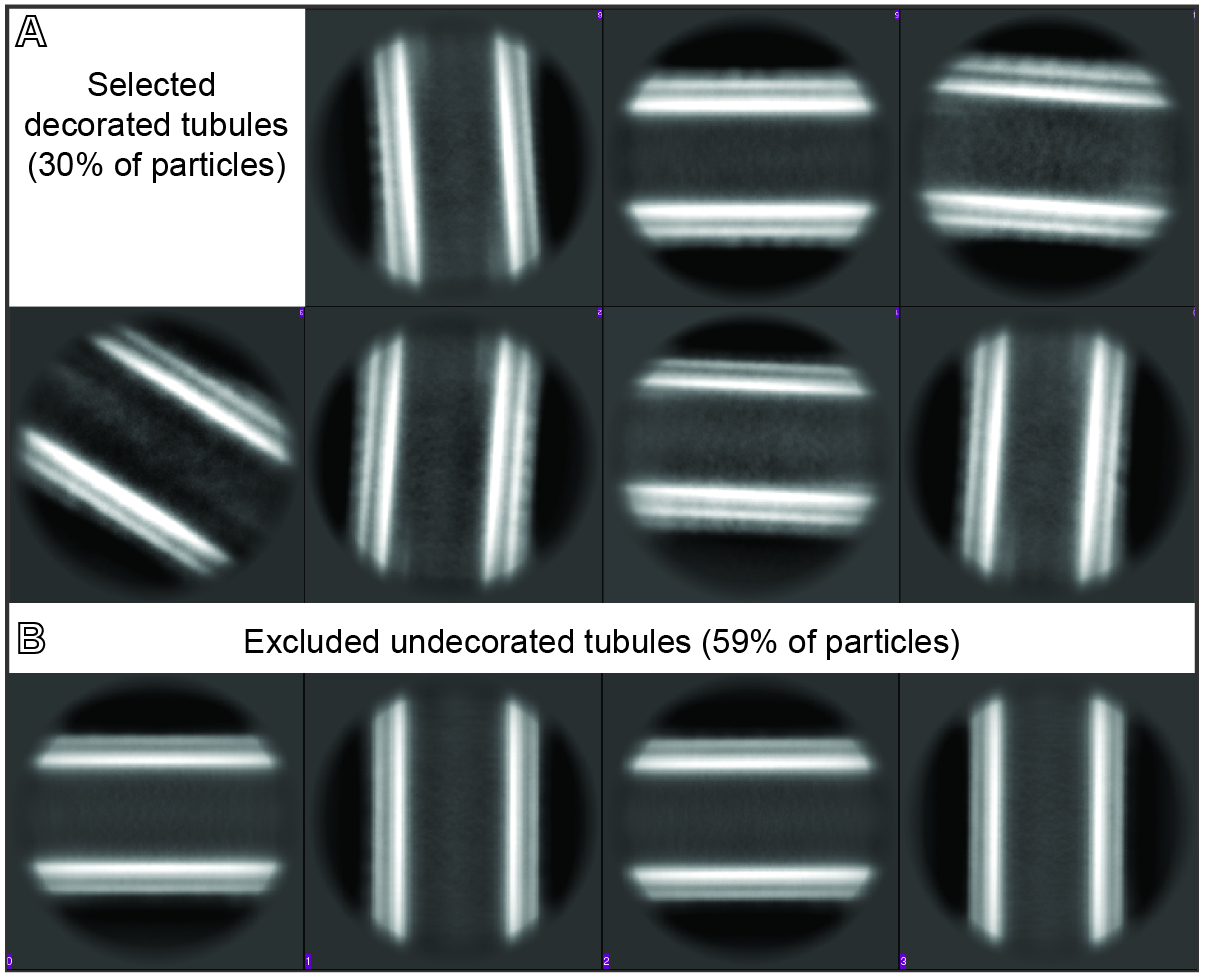


S. Figure 9. 2D Classification of Sar1∙∙∙GalCer. A 220,762 particles were classified, and 64,170 decorated particles were selected as containing decorations. Ordered decorations have discrete densities on the outside of the membrane, while disordered density is amorphous. B Undecorated tubules present as bare, with no density outside of the membrane.
